# The ‘Criaderas and Solera’ System in Sherry Wines: Biological Aging, Flor Yeast Dynamics, Industrial Applications and Emerging Challenges—A Review

**DOI:** 10.3390/foods14244211

**Published:** 2025-12-08

**Authors:** Juan C. García-García, Juan C. Mauricio, Teresa García-Martínez, Juan Carbonero-Pacheco

**Affiliations:** Department of Agricultural Chemistry, Edaphology and Microbiology, Agrifood Campus of International Excellence ceiA3, Universidad de Córdoba, 14014 Córdoba, Spain; p22gagaj@uco.es (J.C.G.-G.); mi2gamam@uco.es (T.G.-M.); b12capaj@uco.es (J.C.-P.)

**Keywords:** sherry wine, criaderas and solera, flor yeast, biological aging, *Saccharomyces cerevisiae*, biofilm formation, acetaldehyde, wine biotechnology

## Abstract

The Criaderas and Solera system represents one of the most sophisticated aging methodologies in winemaking, producing distinctive Sherry wines through dynamic blending and biological aging processes. This traditional Spanish system, combined with the unique metabolic activities of flor yeast (*Saccharomyces cerevisiae*), creates wines of exceptional complexity and consistency. This comprehensive review synthesizes current literature on the Criaderas and Solera system, focusing on biological aging mechanisms, flor yeast dynamics, industrial applications, and contemporary challenges. Recent advances in genomic, proteomic and metabolomic studies of flor yeasts have been analyzed, alongside modern analytical approaches to the characterization of Sherry wine. The Criaderas and Solera system enables consistent wine quality through fractional blending across multiple aging levels. Flor yeasts exhibit specialized metabolic adaptations, including enhanced ethanol oxidation capacity, biofilm formation abilities, and stress resistance mechanisms. Modern applications extend beyond traditional winemaking to include biotechnological uses, immobilization techniques, and sustainable production methods. Current challenges include climate change impacts, maintaining genetic diversity, adapting to new technologies and meeting evolving consumer preferences. The integration of traditional knowledge with modern biotechnology offers promising opportunities for optimizing Sherry production. Understanding flor yeast ecology and metabolic pathways provides insights for developing innovative applications while preserving the authenticity of this centuries-old winemaking tradition.

## 1. Introduction

The production of Sherry wines represents a complex oxidative–biological maturation process characteristic of Southern Spain, integrating nuanced biochemical and technological steps that contribute to their distinctive character. Central to this tradition is the unique “Criaderas and Solera” aging system, which employs fractional blending across multiple barrel scales, maintaining a biological average in each row and facilitating both consistency and gradual evolution in wine profile through generations [1].

This methodology, originating in southern Spain, is intimately linked to the metabolic activities of specialized flor yeasts that form biofilms on the wine surface and mediate biological aging via oxidative metabolism [2,3]. The Criaderas and Solera system is used to produce not only Sherry wines such as Amontillado, Fino, Oloroso and Palo Cortado, but also some naturally sweet wines such as Moscatel and Pedro Ximénez, as well as brandy and vinegar [1,2].

Although the dynamic aging technique has remained largely unchanged for centuries, it continues to be the subject of ongoing scientific investigation due to its remarkable consistency and ability to produce exceptionally complex wines. Modern studies in genomics, proteomics, and metabolomics have revealed elaborate mechanisms by which flor yeasts adapt to fortified wine conditions and generate key compounds that define Sherry wine quality [3,4,5,6].

The industrial relevance of Sherry production now extends beyond winemaking, impacting areas such as biotechnology, food science, and the pursuit of sustainable production systems [7,8]. Nonetheless, the industry faces contemporary challenges, including fluctuating climate conditions, the need to preserve flor yeast genetic diversity, technological adaptation, and shifting consumer demands [7].

## 2. The Criaderas and Solera System: Traditional Methodology and Modern Understanding

### 2.1. Historical Background and System Architecture

The Criaderas and Solera system, developed in the 18th century, represents a revolutionary approach to wine aging that achieves consistency through complexity. The system typically consists of three to four levels of barrels, with the Solera at the bottom containing the oldest wine, and successive Criaderas above containing progressively younger wines [1,8].

#### 2.1.1. Barrel Organization and Operational Principles

The operational principle involves a series of barrels of 600 L of capacity, disposed in rows, distinguished by their aging periods:

• Solera (bottom tier): Contains the oldest wine; used for commercial bottling.
• Criaderas (middle tiers): Each contains progressively younger wine, numbered sequentially.
• Youngest tier: Filled with current vintage wine.

**Figure 1:**
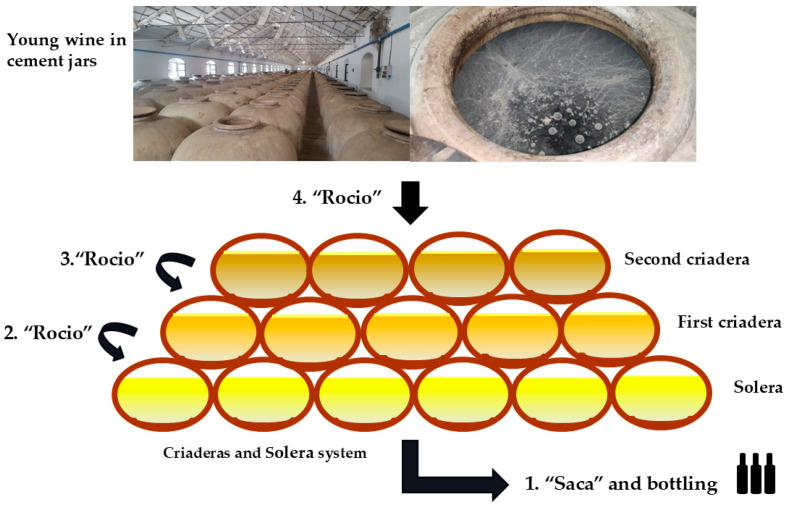
“*Saca* *y rocio*” (removal and replenishment) scheme in a “Criaderas and Solera” system from the Montilla-Moriles PDO. 1, 2, 3, 4: Indicates the order in the process. (Original photographs by authors).

This hierarchical structure is maintained through the “*Saca y Rocío*” process (Figure 1).

##### Saca y Rocío: The Fractional Blending Process

The “*saca y rocío*” (removal and replenishment) process involves three sequential steps:

1. ***Saca*** **(extraction):** Approximately one-third of the wine is extracted from the Solera for commercial bottling.
2. ***Rocío*** **(replenishment):** The Solera is refilled with an equivalent volume from the first Criadera.
3. **Sequential replacement:** Each subsequent row is replenished from the row above, continuing through all tiers until the youngest stage is filled with the current vintage wine (Figure 1) [9].

This systematic transfer preserves wine quality over time, facilitating nutrient exchange and aeration within the system–both essential for maintaining the flor veil (biofilm). Moreover, it enables the homogenization of different vintages, thereby preserving the product’s quality and characteristic profile over the years.

##### Headspace and Oxygen Management

**Figure 2:**
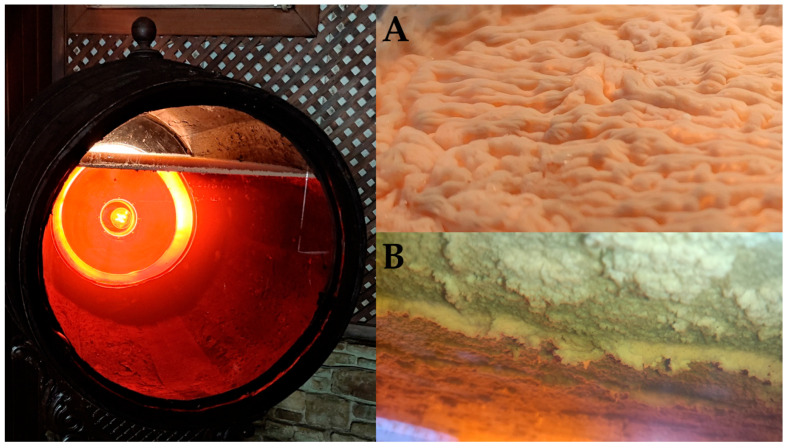
Flor veil (biofilm) on Fino wine surface. (**A**) Detail of the surface exposed to the air. (**B**) Detail of the surface exposed to the wine. (Original photographs by authors).

**Figure 3:**
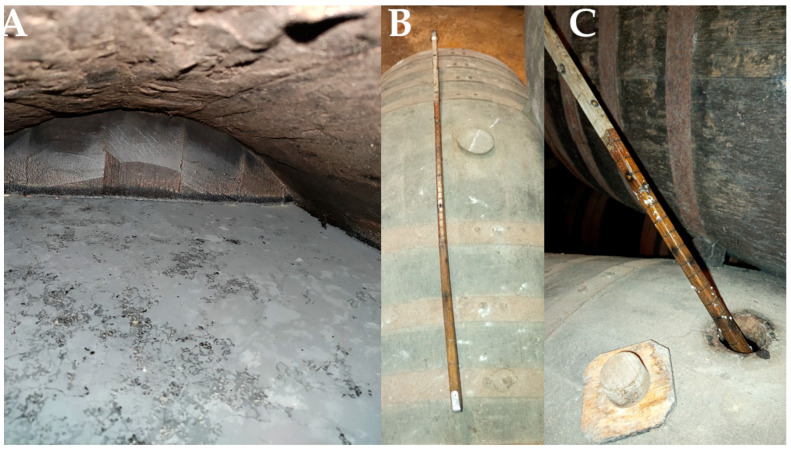
(**A**) Interior of a barrel with approximately 1/6 volume empty, to promote the development of the flor biofilm. (**B**) *Aspilla* (Dipstick). (**C**) Measuring the amount of wine contained in the barrel with a dipstick; traces of the flor can be seen on the dipstick. (Original photographs by authors).

During biological aging, barrels are not filled to its full capacity; instead, approximately one-sixth of the vessel’s volume is left as headspace to facilitate oxygen exchange within the barrel, thereby promoting optimal development of the flor biofilm in the wine surface (Figure 2) [10]. The volume of wine inside the barrels is regularly monitored using a calibrated rod called “*aspilla*” (dipstick), which allows wine’s volume quantification without disturbing the integrity of the flor biofilm (Figure 3).

#### 2.1.2. System Constraints and Economic Trade-Offs

**Figure 4:**
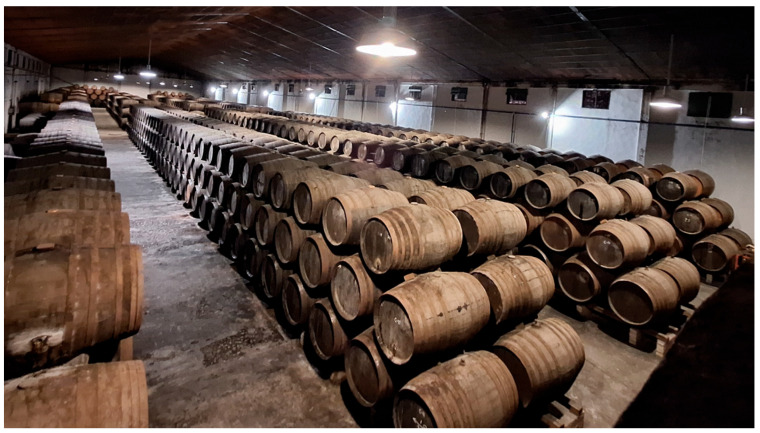
Example of a Criaderas and Solera system in a traditional Andalusian (Spain) winery. (Original photograph by authors).

A significant drawback of this system is the requirement for a substantial amount of wine (approximately two-thirds per barrel) to remain stationary within the barrels and not be commercialized or used to replenish subsequent rows after each “*saca y rocío*” cycle (Figure 4) [11]. This represents a considerable economic and spatial investment, though necessary for maintaining system integrity and product consistency. Therefore, any innovation or improvement in this area would have a significant economic impact.

### 2.2. Fortification and Pre-Aging Stages

#### 2.2.1. Ethanol Elevation Strategies

The alcohol content (%, *v*/*v*) of the wine is maintained at elevated concentrations (above 15%, *v*/*v*) prior to entry into the Criaderas and Solera system to promote flor yeast development and to reduce the risk of contamination by other microorganisms. Such ethanol levels can be naturally achieved during alcoholic fermentation using grape varieties that achieve high sugar concentrations during grape ripening, such as Pedro Ximénez in the Montilla-Moriles Protected Designation of Origin (PDO) [7,11,12]. Alternatively, ethanol concentration can be artificially increased through a process known as “*encabezado*” (fortification) which involves the addition of wine alcohol to reach the desired content. This technique is predominantly used in the Jerez-Xérès-Sherry and Manzanilla–Sanlúcar de Barrameda PDOs, where the Palomino grape variety, combined with milder climatic conditions, does not naturally achieve 15% (*v*/*v*) alcohol concentration [11].

#### 2.2.2. Regional Variations in Pre-Aging

**Figure 5:**
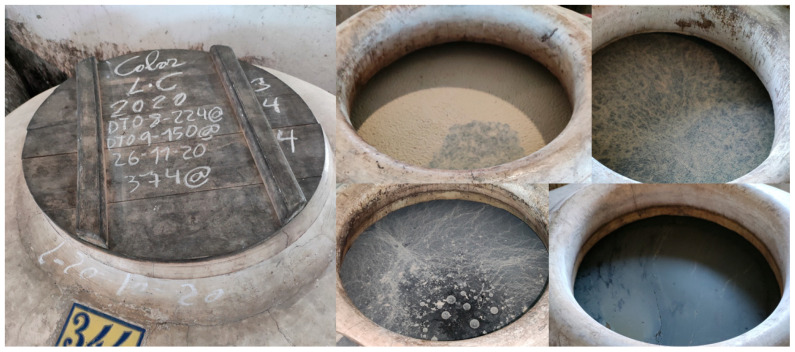
Different flor veils formed in the surface of the wine contained in the cement jars. (Original photographs by authors).

In the Jerez-Xérès-Sherry and Manzanilla–Sanlúcar de Barrameda PDOs: The fortified base wine undergoes a static phase known as “*sobretablas*,” during which the wine is aged in 600 L oak barrels and biological aging is initiated [2,11]. However, in the Montilla-Moriles PDO: The freshly fermented, unfortified wine is stored in large-volume (2000 to 10,000 L) clay or cement jars, where it stabilizes before entering the Criaderas and Solera system [12,13]. During this phase, flor yeast strains develop on the wine surface, forming heterogeneous flor veils (Figure 5). This process induces compositional changes that produce a unique product, promoting its commercialization as a non-aged white wine distinctive to this PDO called “*vino de tinaja*” (jar wine) [12].

### 2.3. Chemical Evolution During Aging

#### 2.3.1. Overview of Chemical Transformations

The chemical transformations occurring within the Criaderas and Solera system are complex and multifaceted, involving both biological and physical-chemical processes [1,2]. Significant compositional changes occur across multiple compound classes throughout the system, including organic acids such as acetic, tartaric, malic, lactic, citric and succinic acids, as well as volatile compounds. Examples of volatile compounds include higher alcohols (such as isoamyl alcohol and isobutanol), aldehydes (such as acetaldehyde), esters (such as ethyl acetate) and phenolic compounds. While organic acids are non-volatile and primarily contribute to taste and acidity, volatile compounds are responsible for the development of aroma and flavor profiles in wine [1,2,8,10,14,15,16].

During biological aging, both pH and total acidity decrease across the Criaderas and Solera system. This trend is partly due to the consumption of organic acids by flor yeast, alongside the precipitation of salts such as potassium bitartrate (KHT). At low pH, KHT precipitation can promote the release of protons (H^+^), further contributing to the observed drop in pH and acidity [17].

##### Acetaldehyde Dynamics

Acetaldehyde concentrations, the characteristic and key compound of biological aging, increase progressively through the system levels, with the highest concentrations found in the Solera [2,15]. This compound is generated from ethanol via the action of alcohol dehydrogenase II, subsequently oxidized to acetic acid, and converted into acetyl-CoA to yield energy [15,16]. Acetaldehyde plays a significant role in enhancing aromatic complexity and imparts a distinctive ripe apple aroma characteristic of Fino wine [18]. This compound is precursor for other volatile compounds of high sensory relevance, including acetoin, 1,1-diethoxyethane, sotolon and 2,3-butanediol [16].

##### Alternative Carbon Sources

Beyond ethanol, flor yeasts metabolize additional carbon sources typically produced during alcoholic fermentation, such as glycerol and organic acids like acetic and gluconic acids [19,20]. Higher alcohols, particularly isobutanol and isoamyl alcohols, are produced through yeast metabolism and contribute significantly to the wine’s aromatic profile [21,22].

##### Nitrogen Metabolism and Amino Acid Consumption

Flor yeasts can utilize a broad spectrum of nitrogen sources, including various dipeptides [23,24]. L-proline emerges as the principal amino acid consumed during biological aging because it is the most abundant in wine after alcoholic fermentation. Recent studies indicate that nitrogen consumption patterns vary depending on the specific flor-forming strain and the wine’s composition, with some strains even able to modulate the concentration of biogenic amines such as histamine and putrescine [20].

##### Physical Concentration Effects

The reduction in volume that occurs in wine due to the transpiration of water molecules through the pores of the wood is another highly significant process. This phenomenon, known as “*merma* or *cuota de los ángeles*” (evaporation loss and angel’s share), results in the concentration of other compounds present in the wine, particularly those of a non--volatile nature. In the case of Fino, this loss reaches a maximum of approximately 4.5% per year, because of the combined action of the wood and the flor yeast activity [2,25].

### 2.4. Analytical Approaches and Quality Control

#### 2.4.1. Chemometric Modeling and Age Prediction

Modern analytical techniques have revolutionized the understanding and quality control of Sherry wines aged in the Criaderas and Solera system. Chemometric approaches, including Principal Component Analysis (PCA) and Multiple Linear Regression (MLR), have proven particularly effective in characterizing wine age and authenticity [2,18]. Studies have developed predictive models capable of estimating wine age with over 97% confidence using just eight analytical variables. These models represent valuable tools for wineries to monitor and control aging processes, ensuring consistency and authenticity in their products [1,18].

#### 2.4.2. Key Markers of Biological Aging

In the context of biological aging, acetaldehyde and glycerol stand out as the most significant compounds for establishing a kinetic model of the process [18]. However, the concentration of acetaldehyde and its related compounds can be readily altered by various winemaking practices. Moreover, acetaldehyde is a highly reactive and volatile molecule, with a boiling point of 25 °C, which makes its use as a reliable indicator of biological aging time unsuitable [16,18].

Glycerol, on the other hand, is the third most abundant constituent of wine after water and ethanol and is considered the most appropriate compound for developing such a kinetic model. Its progressive decrease closely correlates with flor yeast activity, and its concentration in biologically aged wines is relatively stable and not easily subject to external modifications [14,22,26].

Sotolon is a lactone produced from the reaction between α-ketobutyric acid and acetaldehyde. According to Thuy et al. [27], sotolon formation occurs via a purely chemical mechanism and is influenced by temperature, alcoholic content, and decreasing pH. Sotolon has a high odor impact, imparting nutty, curry, and cotton candy notes. Its odorant activity has been widely reported as a good marker for biological aging in both biologically and oxidatively aged wines [28,29].

## 3. Biological Aging: The Role of Flor Yeast

### 3.1. Flor Yeast Diversity and Population Dynamics

#### 3.1.1. Classification and Identification Methods

**Figure 6:**
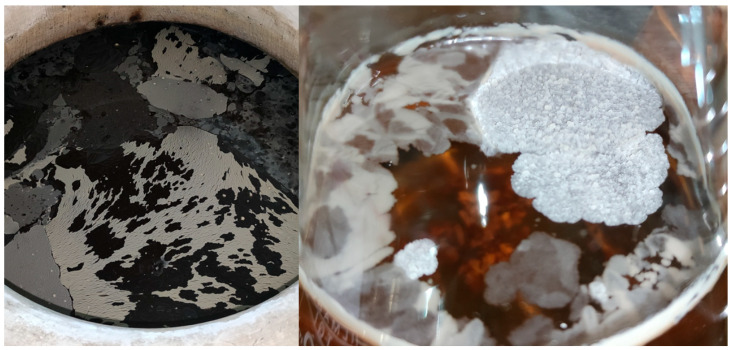
Different veils by flor forming yeast strains growing in wine surface. (**Left**) Cement jar in winery. (**Right**) Erlenmeyer flask in laboratory conditions. (Original photographs by authors).

The phenotypic variability of flor veils and the diverse organoleptic characteristics that they promote in the wine have attracted the attention of both enologists and researchers (Figure 6). Traditional classification approaches preceded modern molecular methods:

Phenotypic Classification (Historical): In Jerez-Xérès-Sherry PDO, flor yeast (*Saccharomyces cerevisiae*) was historically classified into four races (beticus, cheresiensis, montuliensis, and rouxii), based on their ability to metabolize different carbon and nitrogen sources [21,30]. However, this technique presented limitations in the identification accuracy.

Molecular Identification Methods (Modern): The accuracy issue was solved with the use of molecular methodologies including:

• Amplification of the ITS1 (internal transcribed spacer 1)-ITS4 region and the 5.8S-ITS and interdelta regions.
• Isolate analysis via matrix-assisted laser desorption/ionization time of flight mass spectrometry (MALDI-TOF) [3,5,6,11,31].

#### 3.1.2. Species Diversity in Sherry-Type Systems

The dominant flor-forming microorganisms are *S. cerevisiae* strains adapted to biological aging conditions.

The molecular techniques have enabled the identification of diverse *S. cerevisiae* strains and non-*Saccharomyces* yeasts like *Candida guillermondii*, *Pichia kudriavzevii*, *P. manshurica*, *P. membranaefaciens*, *Trichosporon asahii* or *Wickerhamomyces anomalus* in the Criaderas and Solera system [6,32,33].

In Montilla-Moriles PDO, Guijo et al. [34] isolated five flor yeast *S. cerevisiae* strains from flor veils. One notable strain forms a thick, rough film and produces less acetaldehyde, while another forms a thin biofilm and produces large amounts of this aldehyde [35]. Guijo et al. [34] also isolated *Torulaspora delbrueckii* and *Zygosaccharomyces bailii*, which were deemed contaminants, from flor veils. Some authors have additionally isolated species of the genera *Dekkera* and *Brettanomyces*, which are believed to cause an abnormal acidity to increase in casks containing biologically aging wines [36].

The origin of non-*Saccharomyces* yeast species remains unclear; hypotheses include introduction from the “*sobretabla*” phase or contamination within the Criaderas and Solera system [11].

#### 3.1.3. Arthropod Vectors in Microorganism Dispersal

More recent studies in the Montilla-Moriles PDO suggest a novel mechanism for microbial dispersal: the presence of arthropods in the cellar environment. Two mite species (*Carpoglyphus lactis* and *Tyrophagus putrescentiae*) and flies of the genus *Drosophila* may be crucial in disseminating these yeast species [13]. These organisms have previously been associated with acetic acid bacteria and a broad yeast diversity, including the genera *Candida*, *Pichia* and *Kluyveromyces* [37,38].

##### Mechanism of Dispersal

**Figure 7:**
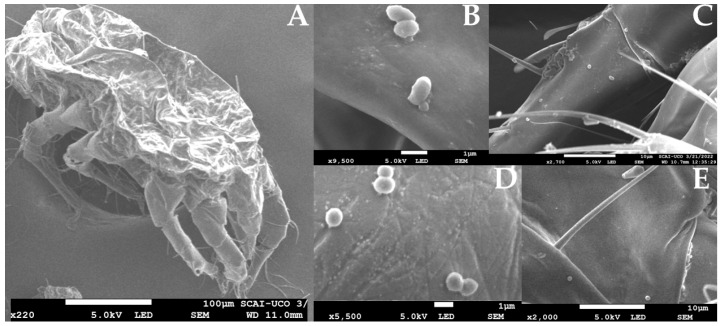
Scanning Electron Microscope (SEM) images. (**A**) *Carpoglyphus lactis* mite; (**B**–**E**) different microorganisms morphologies detected on mite exoskeletons. Source: original, mites samples were fixated, prepared and photographed with a JSM-7800 F Field Emission SEM (JEOL^®^) as indicated in [13].

The results indicate that mites feed on the flor veil at various stages of the Sherry wine production process. They actively move throughout the cellar, transporting microorganisms, including yeasts, on their bodies–from cement jars to the solera barrels [13]. This suggests a potential vector role of cellar arthropods in introducing and distributing heterogeneous microbial populations to the biological aging process (Figure 7) [13].

##### External Biofilm Formation: “Nata” (Cream)

**Figure 8:**
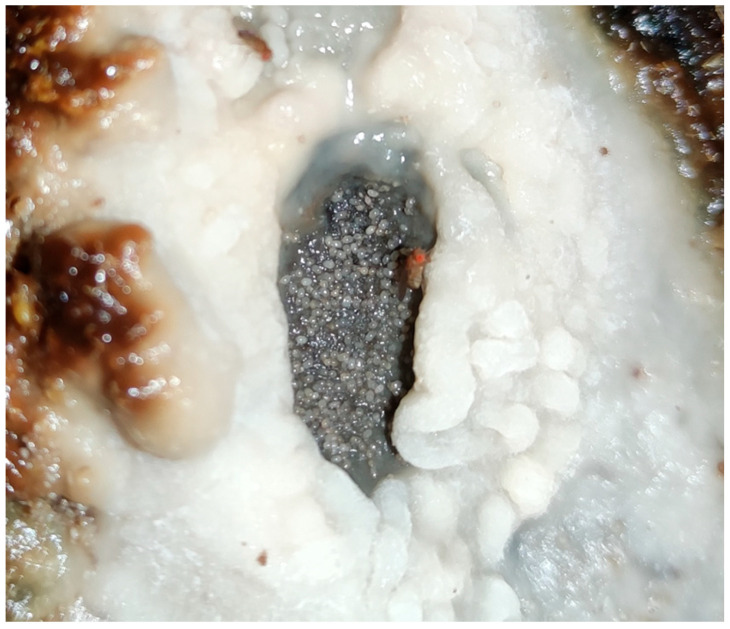
“*Nata*” (cream) formed around a “*salidero*” (wine seep or leakage spot). Mite aggregations could be observed in the biofilm together with *Drosophila*-like flies. (Original photograph by authors).

Mites and *Drosophila* flies have been described as contributors to the formation of an external biofilm on the barrel surface, known as “*nata*” (cream). This biofilm has been found to harbour up to 22 distinct microbial species, identified using MALDI-TOF mass spectrometry and ITS gene amplification techniques (Figure 8) [13].

The presence of acetic acid bacteria and the yeast species *Brettanomyces bruxellensis* among the microorganisms identified in the ‘*natas*’, as well as the possibility that mites and *Drosophila* flies could be vectors of these microorganisms, may pose a risk to the microbiological stability of the flor veil. This has implications for hygiene and health management. Further studies are therefore required to evaluate these aspects.

##### Current Knowledge Gaps

Microorganisms associated with mites are widely studied due to their potential as food contaminants and/or human pathogens [39,40]. It is known that *C. lactis* serves as a transport agent of yeast and bacteria during its feeding activity in other environments or substrates such as dried fruits, milk, cheese or jam [41]. However, no available studies address the ability of these arthropods to modulate the microbiota in wine or in the flor biofilm.

### 3.2. Genomic and Proteomic Insights

#### 3.2.1. Chromosomal Architecture and Adaptive Genetics

Flor yeasts exhibit pronounced variability in both nuclear and mitochondrial genomes [21,30,42,43]. Genetic studies have documented: Extensive aneuploidies (abnormal chromosome copy numbers). An unusually high number of supernumerary copies of specific chromosomes. Chromosome XIII as particularly notable, containing genes encoding alcohol and aldehyde dehydrogenases involved in the metabolism of ethanol, acetaldehyde, and acetic acid [35,44,45,46,47].

These aneuploidies, together with chromosomal rearrangements, confer enhanced resilience to the physicochemical conditions prevailing during wine aging and contribute significantly to sexual isolation [48]. Such isolation constrains the random redistribution of advantageous traits, with most flor strains displaying markedly reduced sporulation and yielding meiotic products that are frequently inviable [43,49].

#### 3.2.2. Molecular Markers for Flor Yeast Identification

Analyses of the internal transcribed spacers (ITS) within the 5.8S rRNA gene have revealed a characteristic 24 bp deletion in *S. cerevisiae* flor strains. This deletion serves as a potential molecular marker for their reliable identification and authentication [50,51].

#### 3.2.3. Strain-Specific Stress Tolerance

Aranda et al. [52] demonstrated that flor yeast strains isolated from wines in the solera row exhibited greater resistance to acetaldehyde and ethanol than those from other scales. This enhanced stress tolerance was positively correlated with elevated transcription levels of heat shock protein (HSP) genes [52].

#### 3.2.4. Genomic Adaptation Landscape

Whole-genome sequencing of flor yeast strains has revealed a complex landscape of genetic adaptations distinguishing them from conventional wine yeasts. Analysis of 2270 genetic variants in 1337 loci specific to flor yeast strains has identified changes related to: Cell morphology, Mitotic cell cycle regulation, Ion homeostasis, and Carbohydrate metabolism [3,43].

#### 3.2.5. Proteomic Analysis of Biofilm Formation

Proteomic studies have identified key proteins involved in biofilm formation, with particular emphasis on respiration, translation, stress damage prevention, and amino acid metabolism. Proteins such as Bgl2p, Gcv3p, Hyp2p, Mdh1p, Suc2p, and Ygp1p have been quantified at very high levels during biofilm formation, providing insights into the molecular mechanisms underlying veil development [4,53].

### 3.3. Flor Yeast Biofilm Formation During Biological Aging

#### 3.3.1. Environmental and Chemical Requirements

The formation, thickness, appearance, and color of the flor veil are determined by multiple factors, particularly in *S. cerevisiae* flor strains. Environmental conditions exert major influence on biofilm development [11]:

Temperature: The optimal temperature for biofilm development lies between 15 °C and 22 °C, with an acceptable range of 15–20 °C (Table 1). Temperatures exceeding 22.5 °C markedly increase the frequency of respiratory-deficient (rho^−^) mutants incapable of forming a flor film [42].

Humidity: Relative humidity above 70% is also essential to sustain proper biofilm formation.

Oxygen: Oxygen concentration strongly affects both the rate and thickness of the film [22,54].

Ethanol: Ethanol concentration constitutes another critical factor; with optimal flor development occurring in wines containing 14.5–15.5% (*v/v*) of ethanol; above 16.5% (*v/v*), biofilm formation is rarely observed [2].

Residual Sugars: The role of residual sugars remains controversial: some authors report that concentrations of 1–1.6 g/L are necessary for biofilm development, whereas others have found no significant effect [35].

Nitrogen Availability: During the biological aging process, nitrogen needs of flor yeasts are largely met through the aerobic metabolism of L-proline, which is converted into glutamic acid, promoting biofilm growth and persistence [23,54,55].

Additional compounds contributing to flor veil formation include phenolic substances, biotin, and pantothenate [56].

**Table 1:** Thermal comfort ranges recommended by different authors for biological aging of Fino wine. This is recorded by Cañas et al. [57].

Author	Limit Range	Optimal Range
Marcilla	10–25 °C	15–17 °C
Fernández de Bobadilla	-	15–20 °C
Cruess	Not reported–26.6 °C	Not reported–20 °C
García del Barrio	12–25 °C	-
Lozano and Perdigones	-	18–20 °C
Barbadillo	-	16–18 °C
Bravo Abad	Not reported–25 °C	15 °C
Yravedra-Soriano	-	12–22 °C
Suárez Lepe and Íñigo Leal	13–25 °C	15–18 °C

#### 3.3.2. Molecular Mechanisms of Biofilm Formation

##### Morphological and Physiological Adaptations

Flor veil formation represents an adaptive response of *S. cerevisiae* to the harsh conditions present in biologically aged wines, including: Elevated ethanol and acetaldehyde concentrations, oxidative stress, and limited nitrogen sources such as L-proline [20].

This adaptation involves morphological and physiological changes—alterations in cell size, shape, and surface hydrophobicity—that lower cell density and enable flotation [24].

Transition to the film-forming state is accompanied by increased levels of long-chain fatty acids, particularly a higher C18:1/C18:0 ratio, which enhances ethanol tolerance and surface hydrophobicity [24,58,59,60].

Hydrophobicity arises from the expression of surface hydrophobic proteins, as treatment with proteinases inhibits biofilm formation [61]. Although the molecular mechanism remains complex and not fully elucidated, biofilm formation occurs when flor yeasts utilize ethanol as an aerobic carbon source and is repressed by glucose [24,62,63,64,65,66,67,68,69].

##### The FLO11 Gene

The gene *FLO11*, encoding a hydrophobic cell wall glycoprotein, is crucial for biofilm formation and confers buoyant density surpassing that of the surrounding medium [24,67]. Regulation of *FLO11* by *NRG1* enhances biofilm development and increases cell surface hydrophobicity [63]. This increased hydrophobicity enables yeast cells to float via surface tension, granting access to atmospheric oxygen and facilitating survival in oxygen-limited wine environments [24,64,68,69,70].

##### Metabolic Shifts and Gene Expression

The metabolic shift from fermentative to oxidative pathways constitutes a key adaptation that supports flor yeast survival during biological aging [45]. Transcriptomic studies reveal dynamic gene expression changes throughout veil formation, including upregulation of genes involved in oxidative carbon metabolism, high-affinity sugar transport, and stress response [5,21].

Notably, *FLO11* expression is strongly elevated during mature biofilm stages, while other flocculin genes remain unchanged or downregulated, indicating a unique role of *FLO11* in the air-liquid interface biofilm characteristic of flor yeasts [3,4,5,24].

## 4. Applications and Biotechnological Innovations

### 4.1. Traditional Applications Beyond Sherry Wine

#### 4.1.1. Geographic and Varietal Expansion

Flor yeast and biological aging have recently been applied to a wider array of grape varieties beyond the original Palomino and Pedro Ximénez varieties and wine regions, demonstrating versatility beyond their classic contexts [8]. Notably, flor yeast has been utilized for biological aging in grape varieties such as Savagnin in Jura, France—central to Vin Jaune production—as well as Airén, Sauvignon Blanc, and selected red varieties, producing wines with distinctive oxidative profiles and complex aromas [8,31].

This approach to flor aging has been adopted in various wine regions including Jura, Russia, Australia, and parts of Italy, where biofilm-forming yeasts have been integrated with local winemaking traditions to produce unique styles [8].

Beyond traditional Sherry, flor yeasts are increasingly employed in diverse fermented beverages and spirits, owing to their ability to form robust biofilms and metabolize ethanol aerobically [7]. These features facilitate innovations such as lower-alcohol wines and novel sensory profiles derived from diverse grape varieties.

#### 4.1.2. Novel Applications in Red and Sparkling Wines

Red Wine Aging: Flor aging, though less typical for red wines, has been shown to induce oxidative, nutty aroma notes while diminishing fruity characteristics and altering mouthfeel, modulating key parameters such as ethanol content, astringency, and color [71].

Sparkling Wine Innovation: Recent advances include the application of the indigenous *S. cerevisiae* G1 strain from Montilla-Moriles PDO for second fermentation in sparkling wine production. This strain is now used commercially by a wine company and has yielded promising results alongside strain N62 [72,73,74].

Metabolomic profiling revealed significant differences between the strains: G1 produced elevated levels of 3-methyl-1-butanol, 2-methyl-1-butanol, and acetaldehyde, whereas N62 generates higher concentrations of glycerol, ethyl esters, and amino acids such as glutamic acid, aspartic acid, and alanine. These differences highlight the potential of these strains to impart unique sensory characteristics and diversify sparkling wine products [72,73,74].

#### 4.1.3. Non-Saccharomyces Applications Isolated from Flor Veil

The non-*Saccharomyces* yeast *Wickerhamomyces anomalus*, isolated from Montilla-Moriles flor veil, has been selected for improving the sensory profile of young white wines. Studies confirm its successful implantation and its capacity to produce high ethyl acetate and lower ethanol levels [33].

The exploitation of flor yeast continues to broaden the horizon of biological aging, enriching the diversity and sensory appeal of wines worldwide [8].

### 4.2. Yeast Immobilization Technologies

#### 4.2.1. Co-Immobilization with Filamentous Fungi

*Saccharomyces cerevisiae* strain G1, known for its robust flor yeast characteristics, shows excellent performance when immobilized, especially through co-immobilization with filamentous fungi such as *Penicillium chrysogenum*. This immobilization maintains the strain’s metabolic activity and viability, enabling repeated use in fermentation, particularly in sparkling wine production [75].

These approaches have been successfully applied in food and beverage fermentations and bioethanol production, demonstrating broad biotechnological potential with improved operational stability and sustainability [76,77,78]. Recent proteomic analyses reveal that immobilized G1 cells exhibit enhanced stress response mechanisms at DNA, RNA, and protein levels compared to free cells, which supports greater fermentation stability and potential for modulating sensory profiles [79].

The natural yeast-fungal biocapsule system offers a cost-effective and sustainable immobilization method, improving process efficiency and expanding applications beyond traditional winemaking into various food fermentation technologies. This positions strain G1 as a promising candidate for industrial fermentation processes requiring enhanced control, product consistency, and diversification.

#### 4.2.2. Vacuum Infusion Immobilization

Recently, this technology has advanced notably with improved methodologies such as vacuum infusion with *Aspergillus oryzae*, which enhances yeast cell loading, retention, and viability within fungal pellets, leading to superior biocapsule productivity and fermentation performance compared to traditional co-culture approaches [80]. These immobilized systems find extensive use in intensified fermentations, particularly in winemaking including sparkling and Sherry wines, where they help modulate wine aroma, sensory profiles, and process consistency [7].

#### 4.2.3. Low-Alcohol Sherry Production

Recent advances in yeast immobilization have opened new possibilities for Sherry wine production. The development of microbial biocapsules for flor yeast immobilization represents a significant technological advancement, enabling the production of high-quality differentiated Sherry wines without traditional fortification. These immobilization systems offer several advantages, including reduced ethanol consumption, improved process control, and the potential for producing lower-alcohol Sherry-style wines meeting contemporary market demands.

The technology has shown promising results in microvinification trials, with sensory panels evaluating the resulting wines positively [7].

### 4.3. Biotechnology and Industrial Fermentation

#### 4.3.1. Physiological Basis for Industrial Applications

The ability of flor yeast to form biofilms and its unique metabolism make it an interesting model for physiological, molecular evolution and genetic stress studies [15,43]. Due to the extreme conditions in which the yeast strains are found–the absence of fermentable sugars, the scarcity of nitrogen sources, the high concentration of ethanol and acetaldehyde, and their genetic and metabolic characteristics–make them attractive candidates for various industrial fermentation applications [24,70].

Their ability to withstand high ethanol concentrations, form stable biofilms, and produce specific metabolites has applications in biofuel production, pharmaceutical fermentations, and specialty chemical synthesis [3].

#### 4.3.2. Strain-Specific Optimization of Biological Aging

The isolation and characterization of specific strains can be highly valuable for optimizing the biological aging process. This optimization may involve accelerating glycerol consumption, enhancing acetaldehyde production, or modulating the levels of undesirable compounds such as biogenic amines [7,20].

Morales et al. [15] studied the impact of cultivating 15 different flor veil yeast strains in pure culture over one month of biological aging. They observed significant differences in the volatile metabolites produced. Notably, most acetals increased, with acetaldehyde diethylacetal showing the most substantial rise. In addition, terpenes such as nerolidol and farnesol exhibited remarkable increases.

#### 4.3.3. Stage-Specific Strain Selection During Aging

Other studies in the Montilla-Moriles PDO suggest that each flor yeast strain metabolizes wine differently. Depending on the type of wine and the stage within the Criaderas and Solera system, certain strains may be better suited for specific phases of biological aging. This research provides important insights that could help improve traditional aging practices by employing specific yeast strains tailored to each stage of the biological aging process [20].

#### 4.3.4. Genetic Improvement Strategies

Genetic manipulation strategies, including hybridization and adaptive laboratory evolution, have been employed to improve industrial flor yeast strains, particularly in reducing undesirable metabolite production such as hydrogen sulfide [3]. These approaches demonstrate the potential for developing specialized strains tailored to specific industrial applications.

## 5. Emerging Challenges and Future Perspectives

### 5.1. Climate Change Impacts

#### Temperature and Humidity

Climate change presents considerable challenges to the Sherry wine industry, notably impacting grape cultivation, fermentation, and aging processes. Rising temperatures and shifting precipitation patterns disrupt the delicate conditions required for optimal flor yeast activity, essential for the traditional biological aging characteristic of Sherry wines [81].

Adaptation strategies being investigated include the development of climate-resilient grape varieties, modified viticultural practices, and adjusted winemaking protocols. The industry is also exploring precision agriculture technologies and renewable energy systems to enhance sustainability and resilience [82].

**Figure 9:**
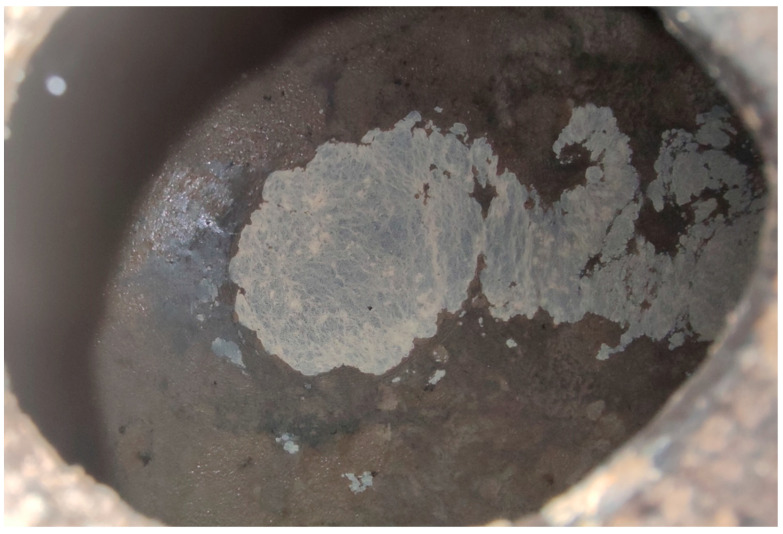
Flor yeast on the surface of Fino wine in deteriorated condition after the exposure to elevated temperatures during summer; an incomplete development of the biofilm is observed in the centre, whereas the peripheral areas display a darkened and degraded flor structure. (Original photograph by authors).

High temperatures and low relative humidity can reduce the stability of the yeast veil. This increases the risk of losing the veil, which protects against oxidation and deterioration of the wine’s sensory quality. It also makes it more likely that the wine will become contaminated by acetic bacteria and other undesirable microorganisms (Figure 9).

To counter these effects, research efforts focus on climate-resilient vineyard management, advanced winery designs with climate control systems—even considering their high energy costs—and innovative bio-based solutions. Among these, the addition of bee pollen provides yeast assimilable nitrogen to support flor growth; meanwhile, 3D-printed supports made from polylactic acid physically stabilize the biofilm [83,84,85]. The authors’ experimental use of polyurethane foam combined with selected thermotolerant yeast strains also aims to mitigate the effects of climate-induced stress on biological ageing.

These integrated strategies seek to preserve the flor veil integrity and ensure the continued quality and traditional character of Sherry wines amid increasing climatic pressures.

### 5.2. Genetic Diversity Conservation

Maintaining the genetic diversity of flor yeast populations represents a critical challenge for ensuring the continued authenticity and quality of Sherry wines. Long-term cultivation in industrial conditions has the potential to reduce genetic diversity, though studies have shown remarkable genetic stability in some industrial strains over decades [3,5,86].

To address this, systematic screening and conservation programs are being developed to identify and preserve promising flor yeast strains from natural populations. These efforts combine molecular genetic markers with physiological and oenological assessments to ensure comprehensive strain characterization and selection [8,87,88,89].

### 5.3. Sustainable Production Systems

#### 5.3.1. Environmental and Operational Pressures

The industry faces increasing pressure to develop more sustainable production systems that minimize environmental impact while maintaining product quality. This includes reducing water usage, minimizing chemical inputs, implementing renewable energy systems, and developing circular economy approaches to waste management [86,89].

Innovation in packaging, distribution, and consumer engagement represents additional opportunities for sustainability improvements. The integration of precision viticulture technologies, including remote sensing and artificial intelligence, offers potential for optimizing resource usage while maintaining wine quality [8].

#### 5.3.2. System Limitations and Accelerated Aging Tecnologies

**Figure 10:**
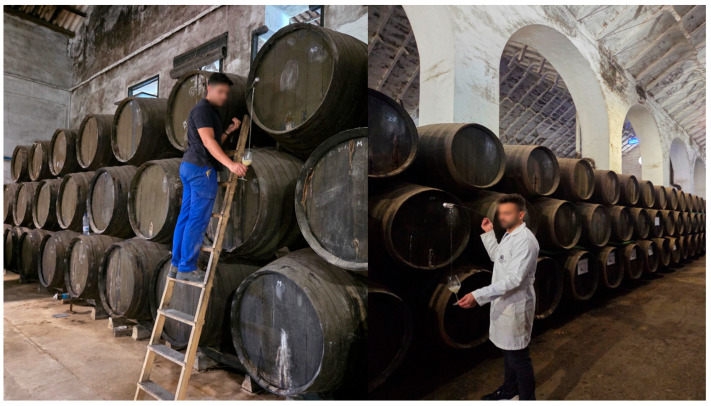
Winery technicians conducting an organoleptic taste of wine from each barrel of a “Criaderas and Solera” system prior to the “*saca y rocío*” process. (Original photographs, taken with the permission of the people in the photograph).

In terms of barrel management, the use of the Criaderas and Solera system presents several limitations. Firstly, the need for a large number of barrels involves considerable labor, both in the sensory evaluation processes, which must be conducted individually (Figure 10), and in the operations of “*saca y rocío*”. Furthermore, this system entails a very prolonged aging period and a substantial economic impact due to “*merma*” (loss during aging), which can reach up to 4.5% in each barrel per year [2].

For these reasons, some collaborative efforts between wineries within the Montilla-Moriles PDO and research groups have focused on developing a device capable of accelerating the biological aging of Sherry-type wines [90].

##### Accelerated Biological Aging Device

The purpose of this device is to shorten the biological aging time of wines undergoing biological aging by supplying controlled amounts of atmospheric oxygen. Two factors have a powerful influence on the duration of the biological aging process: dissolved oxygen level and temperature.

These two variables are controlled indirectly and very imprecisely in the traditional system through the construction of cellars that buffer climatic changes, such as semi-basements, basements, very high ceilings or air chambers, as well as the type and thickness of the wood used to make the barrels, which leads to greater or lesser porosity. In contrast, the device permits precise control of these parameters using specific sensors connected to a computer, allowing them to be maintained at preset values and changed to best suit different types of wines, thereby accelerating the aging process.

The result of its application is a shortening of the aging time, obtaining in laboratory conditions acetaldehyde and glycerol content similar to that of a 5-year-old fino wine in a period of 154 days [90].

The device consists of a stainless-steel container with different probes in which there is a system for circulating the wine from the bottom to an external aeration chamber. The aerated wine is returned to the container through a tube, preventing the flor veil disruption. This allows for controlled aeration and speeds up the process; however, an excessive decrease in ethanol content (below 14%) occurs under these conditions, therefore some improvements are needed before its industrial scale up [90].

### 5.4. Market Adaptation and Consumer Preferences

#### 5.4.1. Shift Toward Lower-Alcohol Wines

Evolving consumer preferences, particularly the shift toward lower-alcohol and sustainable wine options, pose both challenges and opportunities for traditional producers of biologically aged wines in Andalusia, Spain. The development of production methods for reduced-alcohol Sherry wines using modified flor yeast systems represents one approach to addressing these market demands [82].

##### Technical Feasibility of Reduced-Alcohol Production

Recent works have developed practical innovations to help the sherry industry adapt to new market needs while maintaining sensory authenticity and regulatory compliance [91,92]. Pilot-scale studies have demonstrated the technical feasibility of producing Fino-style wines with an ethanol concentration reduced to around 14% (*v*/*v*), while maintaining the distinctive chemical and sensory profile typical of traditional fortified versions. This is achieved through controlled modifications of the biological aging process under flor veil, where flor yeasts actively metabolize ethanol during oxidative growth.

##### Microbiological Risks of Ethanol Reduction

While moderate ethanol reduction opens doors to innovation and improved sustainability in the Sherry sector, it demands vigilant microbiological control. Proper monitoring and management are essential to avoid unintended dominance of undesirable yeast species and ensure the faithful maintenance of PDO standards.

Recent research by authors demonstrates that, when carefully managed, flor yeast can remain active at moderate alcohol levels (around 14% *v*/*v*), continuing to produce stable biofilms and desirable sensory profiles typical of Fino wines. However, findings also indicate an increased presence of non-*Saccharomyces* species in younger Criaderas, especially as ethanol concentration decreases [91,92].

While these non-*Saccharomyces* yeasts can introduce some positive aromatic complexity and may help diversify wine profiles, their uncontrolled proliferation poses several risks:

(1) Competitive displacement: They may outcompete or disrupt classic flor populations, threatening the stability of biological aging.
(2) Metabolic byproducts: Unwanted byproducts—such as excessive volatile acidity, undesirable aromas, or spoilage compounds—can arise, compromising wine typicity and quality.
(3) Management difficulties: Some non-*Saccharomyces* strains are more difficult to manage in barrel systems and can prove resistant to routine cellar hygiene, raising the risk of persistent contamination.
(4) System disruption: The delicate balance between yeast activity, acetaldehyde formation, and protection against oxidation may be disrupted, especially if veil integrity is compromised at lower ethanol levels.

#### 5.4.2. Consumer Education and Enotourism

Consumer education is fundamental to ensuring the continued appreciation and market expansion of Sherry wines, whose complexity and unique production systems are often unfamiliar to younger or international audiences.

##### Communication Strategies

Communicating the singular attributes of biologically aged wines—such as the flor yeast veil, the Criaderas and Solera system, and their sensory distinctiveness—requires clear, experiential, and culturally grounded strategies that connect tradition with contemporary values [93].

##### Integrated Approaches

Integrating traditional artisanal production practices with modern traceability, quality assurance, and digital storytelling frameworks enhances transparency and reinforces consumer trust. Such approaches align with evolving preferences for authenticity, sustainability, and regional identity in wine consumption, serving to position Sherry wines as both heritage products and modern lifestyle choices.

##### Wine Tourism as Educational Platform

In this context, wine tourism represents one of the most powerful instruments for consumer education and brand engagement. Winery visits, cultural routes, and immersive tastings allow consumers to experience firsthand the craftsmanship, environmental conditions, and microbial phenomena that define Sherry production. These experiential activities not only foster emotional attachment to the product but also strengthen the global recognition of Andalusian enological heritage. The integration of enotourism with educational narratives—onsite and virtual—can thus serve as a catalyst for the sustainable development and international visibility of the Sherry sector [94,95].

### 5.5. New Technology Adaptation

#### 5.5.1. Digital Transformation and Industry 4.0

The Sherry wine sector, rooted in centuries-old craftsmanship, is now embracing a digital transformation driven by the integration of smart technologies, real-time data systems, and precision oenology tools that enhance both control and sustainability across all stages of production.

##### Internet of Things (IoT) Systems

The implementation of Internet of Things (IoT)-based systems has been particularly transformative, enabling continuous monitoring of fermentation processes, barrel conditions, and flor yeast development without compromising the integrity of the product. These technological innovations are redefining the efficiency, safety, and traceability of the biological aging process while preserving its artisanal value [96].

##### Smart Barrel Technology

A key advancement in this field is the development of smart barrel bungs and sensorized environments designed to monitor environmental and structural parameters directly within the casks. The pioneering “smart cork” and “electronic bung” systems, tested in Andalusian wineries, have demonstrated how real-time monitoring of ullage, humidity, pressure, and absorbance can provide early warnings of barrel leaks, deviations in aging conditions, or abnormal yeast development [96].

Using low-energy microcontrollers (e.g., ESP32) and cloud computing platforms like ThingSpeak, these devices autonomously transmit analytical data on color evolution or flor activity, thus facilitating instant quality control decisions. Their non-invasive design allows direct installation in traditional oak barrels, maintaining the aesthetic and functional requirements of the Criaderas and Solera system [97,98].

##### Fermentation Tank Monitoring

In addition to barrel-level control, IoT devices integrated into fermentation tanks allow real-time monitoring of CO_2_ emissions to track fermentation kinetics and detect stuck fermentations at early stages. These systems employ optical or gas sensors combined with data analytics to automate decision-making and reduce manual sampling [97,98].

##### Environmental Optimization

Similarly, wireless temperature and humidity sensors distributed throughout wineries generate a continuous flow of environmental data that can be analyzed through predictive algorithms to optimize the conditions of biological aging and wine conservation [97,99].

##### Traceability and Digital Infrastructure

At a broader scale, these technologies support digital traceability and predictive maintenance, key aspects for quality assurance and compliance with Protected Designation of Origin (PDO) requirements. The fusion of traditional craftsmanship with Industry 4.0 principles—cloud computing, big data, and artificial intelligence—has given rise to the concept of the “Smart Winery,” in which sensor networks, mobile applications, and integrated databases enable seamless control from vineyard to bottle [96].

Such digital infrastructure not only improves process stability but also reduces environmental impact and operational costs. Automated energy management, minimal resource consumption, and remote supervision align with European Green Deal objectives for sustainable agri-food production.

#### 5.5.2. Artificial Intelligence and Machine Learning

Recent digital tools allow more precise monitoring and prediction of biological aging processes in Sherry wine production, facilitating optimized winery management and quality control. The adoption of digital monitoring and artificial intelligence (AI) represents a significant advance in the modernization of Sherry wineries, enabling the integration of traditional knowledge with contemporary data-driven methods [96,97,98].

Through the synergy between biotechnology, microelectronics, and informatics, winemaking processes are becoming progressively data-driven while maintaining the sensorial and cultural authenticity of this centuries-old tradition.

##### AI Applications in Biological Aging

The growing integration of AI into the Sherry wine industry is revolutionizing how producers monitor, predict, and manage biological aging processes and overall wine quality. Building on the advances of sensor technology and IoT connectivity, AI transforms the massive datasets generated in wineries into actionable insights, improving decision-making precision and production outcomes.

Machine learning algorithms can now model complex biochemical and environmental interactions—such as temperature fluctuations, flor yeast activity, and oxygen dynamics—to forecast deviations or optimize conditions in real time, thereby maintaining consistency across Criaderas and Solera systems [100,101].

##### Quality Assessment and Fault Detection

In the context of quality evaluation, predictive algorithms trained on physicochemical and sensory data are capable of estimating wine quality or detecting possible faults before they become perceptible to human tasters.

Machine learning models, such as random forest and support vector machines, have demonstrated classification accuracies greater than 95% when applied to the identification and quality assessment of Sherry and other fine wines using physicochemical and sensory data. For example, Jain et al. [101] and Chen et al. [102] reported such results using electronic nose and NIR-based datasets for rapid wine fault detection and classification.

Beyond quality prediction, AI-driven image analysis and deep learning algorithms, such as convolutional neural networks (CNNs) and YOLO-based architectures, are being applied to monitor flor coverage patterns, barrel surface conditions, and grape maturity through drone or smartphone-based imaging systems. These tools enable winemakers to assess the vegetative status of vineyards, detect flor irregularities, and adapt management practices before they impact product quality [103].

##### System-Level Integration and Blockchain

At the system level, integrating AI with blockchain and IoT frameworks en-hances traceability, automates record-keeping, and strengthens consumer trust through transparent provenance systems. This combination ensures the authenticity of Protected Designation of Origin (PDO) products like Sherry wines while supporting sustainability certification and carbon accounting metrics [104].

##### Research and Development Integration

In research and development, AI provides new perspectives on microbiological and chemical data integration, allowing the modeling of multifactorial relationships between yeast strains, volatile compound networks, and sensory perception. Multi-omics data processed by machine learning are opening pathways toward intelligent strain selection and adaptive cellar management. For biologically aged wines such as Fino or Manzanilla, these techniques are of great applicability in understanding the dynamic equilibrium between metabolism, oxidation, and aroma generation.

##### Predictive Oenology

Ultimately, the integration of AI promotes a predictive, data-driven oenology, fostering more sustainable, consistent, and innovative production systems. Combining traditional expertise with algorithmic intelligence ensures that the Sherry industry can preserve its heritage while adopting transformative practices that lead to higher precision and global competitiveness [101].

## 6. Future Research Directions

### 6.1. Systems Biology Approaches

The integration of genomics, transcriptomics, proteomics, and metabolomics offers unprecedented opportunities for understanding the complex biological systems underlying Sherry wine production. Systems biology approaches can provide comprehensive insights into flor yeast behavior, wine-microbe interactions, and optimization strategies for production systems [105].

Machine learning and artificial intelligence applications in analyzing complex datasets from Sherry production systems represent promising areas for future research. These approaches can help identify subtle patterns and relationships that may not be apparent through traditional analytical methods [2].

### 6.2. Synthetic Biology Applications

The detailed understanding of flor yeast genetics and metabolism opens possibilities for synthetic biology approaches to strain improvement and novel product development. Targeted genetic modifications could potentially enhance desired characteristics while maintaining the essential features that define Sherry wine quality [3].

However, such applications must carefully balance innovation with the preservation of traditional characteristics and regulatory compliance. The industry’s commitment to authenticity and tradition will likely influence the acceptance and implementation of synthetic biology approaches [8].

### 6.3. Microbiome Engineering

Understanding and potentially manipulating the broader microbial communities associated with Sherry wine production represents an emerging area of research. The interactions between flor yeasts and other microorganisms in the production environment may offer opportunities for optimizing wine quality and process efficiency [9]. Microbiome engineering approaches could potentially enhance the resilience and consistency of flor yeast populations while maintaining their essential characteristics.

Such strategies may be particularly valuable for addressing challenges related to climate change and production system sustainability [82].

## 7. Conclusions

The Criaderas and Solera system represents a remarkable achievement in winemaking technology, combining centuries of traditional knowledge with sophisticated biological processes to produce wines of exceptional quality and consistency. The unique biological aging process, mediated by specialized flor yeasts, creates a complex interplay of metabolic activities that fundamentally transform wine chemistry and sensory characteristics.

Recent scientific advances have provided unprecedented insights into the molecular mechanisms underlying flor yeast behavior and biological aging processes. These studies have revealed the remarkable genetic and metabolic adaptations that enable these yeasts to thrive in the harsh conditions of fortified wine while producing the characteristic compounds that define Sherry wine quality.

The industrial applications of flor yeasts extend far beyond traditional Sherry production, with emerging biotechnological uses demonstrating the versatility and value of these specialized microorganisms. Innovations in yeast immobilization, strain improvement, and process optimization offer promising opportunities for enhancing production efficiency and developing new products while maintaining authenticity.

However, the industry faces significant contemporary challenges, including climate change impacts, genetic diversity conservation needs, and evolving market demands. Addressing these challenges will require continued innovation, sustainable practices, and careful balance between tradition and modernization.

Future research directions, including systems biology approaches, synthetic biology applications, and microbiome engineering, offer exciting possibilities for advancing our understanding and capabilities in Sherry wine production. The integration of traditional knowledge with cutting-edge biotechnology provides a pathway for ensuring the continued success and evolution of this ancient winemaking tradition.

The Criaderas and Solera system, with its unique combination of biological and technological sophistication, serves as a model for how traditional food production systems can be understood, preserved, and enhanced through modern scientific approaches. As the industry continues to evolve, maintaining the delicate balance between innovation and tradition will be essential for preserving the authentic character that makes Sherry wines truly exceptional.

## Data Availability

The original contributions presented in this study are included in the article. Further inquiries can be directed to the corresponding author.
